# Efficacy and tolerability of half-yearly long acting injectable palmitate paliperidone after 12 months follow-up

**DOI:** 10.1192/j.eurpsy.2024.1504

**Published:** 2024-08-27

**Authors:** A. Benito, A. Sanchez-Cabezudo

**Affiliations:** ^1^PSYCHIATRY, SESCAM, TOLEDO; ^2^PSYCHIATRY, SERMAS, MADRID, Spain

## Abstract

**Introduction:**

This retrospective study analysed the clinical efficacy, tolerability and treatment satisfaction of patients who switched from receiving palmitate paliperidone monthly (PP1M) to palmitate paliperidone six-monthly (PP6M) after 12 months of follow-up. A total of 48 patients (31 men and 17 women) with recently diagnosed schizophrenia were included.

**Objectives:**

To assess the clinical efficacy, tolerability and treatment satisfaction in a sample of recently diagnosed schizophrenic patients who switched from receiving palmitate paliperidone monthly (PP1M) to palmitate paliperidone six-monthly (PP6M)

**Methods:**

The sample included a total of 48 recently diagnosed schizophrenic (1-5 years) from three Mental Health units in the province of Toledo (Spain). The inclusion criteria were a diagnosis of schizophrenia (based on the ICD-10 criteria), the start of treatment with Long Acting Injectable Paliperidone Palmitate six-monthly (previously with palmitate paliperidone monthly), and the non-utilization of another neuroleptic treatment. A series of demographic variables were recorded, PANSS scale was used to to identify the presence and severity of psychopathology symptoms and the CGI scale was used to assessed the severity of the symptoms finally time to relapse was measured (primary outcome). The scales were again applied at baseline, 3 and 6 and 12 months after the start of treatment

**Results:**

N=48 patients (31 males and 17 females), with a mean age of 31 years. 4.3 years of evolution of illness. During the follow-up period only 2 patients (4%) relapsed. Results showed an improvement in PANSS (baseline 50.8, 3 months 41.9, 6 months 37.3, 12 months 26.1), likewise and improvement in CGI was observed (baseline 4.1, 3 months 3.4, 6 months 2.9, 12 months 2.5).

In terms of tolerability, no secundary effects were reported after treatment change, suggesting a good safety profile and predictable tolerability of PP6M. Patient satisfaction with treatment also improved over time. The study reports that 87% of patients accepted the switch from PP1M to PP6M, with reasons for switching including reduced frequency of administration and increased comfort. Most patients (95%) received antipsychotic monotherapy.

**Conclusions:**

In conclusion, this study suggests that switching from PP1M to PP6M in patients with recently diagnosed schizophrenia was associated with maintained clinical stability, good tolerability and improved patient satisfaction with treatment. These findings support the efficacy and clinical utility of PP6M as a convenient and effective treatment option for patients with schizophrenia.

**Disclosure of Interest:**

None Declared

